# Impact of basic life-support training on the attitudes of health-care workers toward cardiopulmonary resuscitation and defibrillation

**DOI:** 10.1186/s12913-017-2621-5

**Published:** 2017-09-22

**Authors:** Mostafa A. Abolfotouh, Manal A. Alnasser, Alamin N. Berhanu, Deema A. Al-Turaif, Abdulrhman I. Alfayez

**Affiliations:** 1 King Abdullah International Medical Research Center, King Saud bin-Abdulaziz University for Health Sciences (KSBAU-HS), Riyadh, Saudi Arabia; 2 Postgraduate Training Center, Deanship of postgraduate Education & Academic Affairs, KSAU-HS, Riyadh, Saudi Arabia; 3 Postgraduate Education & Academic Affairs, KSAU-HS, Riyadh, Saudi Arabia

**Keywords:** Automated external defibrillator, Cardiac arrest, Concern

## Abstract

**Background:**

Cardiopulmonary resuscitation (CPR) increases the probability of survival of a person with cardiac arrest. Repeating training helps staff retain knowledge in CPR and in use of automated external defibrillators (AEDs). Retention of knowledge and skills during and after training in CPR is difficult and requires systematic training with appropriate methodology. The aim of this study was to determine the effect of basic life-support (BLS) training on the attitudes of health-care providers toward initiating CPR and on use of AEDs, and to investigate the factors that influence these attitudes.

**Methods:**

A quasi-experimental study was conducted in two groups: health-care providers who had just attended a BLS–AED course (post-BLS group, *n* = 321), and those who had not (pre-BLS group, *n* = 421). All participants had previously received BLS training. Both groups were given a validated questionnaire to evaluate the status of life-support education and certification, attitudes toward use of CPR and AED and concerns regarding use of CPR and AED. Multiple linear regression analyses were applied to identify significant predictors of the attitude and concern scores.

**Results:**

Overall positive attitudes were seen in 53.4% of pre-BLS respondents and 64.8% of post-BLS respondents (χ^2^ = 9.66, *p* = 0.002). Positive attitude was significantly predicted by the recent completion of BLS training (β = 5.15, *p* < 0.001), the number of previous BLS training courses (β = 2.10, *p* = 0.008) and previous exposure to cardiac-arrest cases (β = 3.44, *p* = 0.018), as well as by low concern scores, (β = −0.09, *p* < 0.001). Physicians had significantly lower concern scores than nurses (β = −10.45, *p* = 0.001). Concern scores decreased as the duration of work experience increased (*t* = 2.19, *p* = 0.029).

**Conclusions:**

Repeated educational programs can improve attitudes toward CPR performance and the use of AEDs. Training that addressed the concerns of health-care workers could further improve these attitudes.

## Background

Out-of-hospital cardiac arrest (OHCA) is an international health issue, and the reported survival rates after OHCA vary greatly. The survival of patients with OHCA can be improved by reduction of response times, including early cardiopulmonary resuscitation (CPR), early defibrillation, and early advanced care [1]. CPR and the use of automated external defibrillators (AEDs) are core training components in all life-support courses, including basic life-support (BLS) provider training. To successfully complete a BLS course, a provider must demonstrate the psychomotor skills and cognitive knowledge needed to deliver CPR effectively. The American Heart Association (AHA) and the International Liaison Committee on Resuscitation included BLS in their guidelines in 2000 [2, 3]. CPR and AED training are widespread in all sectors of the health-care profession in Saudi Arabia, and the Saudi Commission for Health Specialties requires health-care providers to renew their certification for licensure [4].

BLS training is a requirement at National Guard Health Affairs (MNG-HA) institutions, and enrollment in advanced resuscitation programs also occurs, dependent on an individual’s scope of practice. MNG-HA in Riyadh provides ~20% of the total BLS training in the Kingdom of Saudi Arabia. At MNG-HA, the BLS course is conducted twice a week, with an average of 24 candidates per course. The Postgraduate Training Center (PTC), an international training center at MNG-HA, had been awarded to reach the Gold Level for the first time with American Heart Association in 2014/2015, with 8183 participants in the Middle East and North Africa [5].

Training duration and skill retention have been the subjects of a prospective, randomized study in which a class length of 2 h was found to be sufficient for retention of skills in CPR and AED for an extended period, provided that re-evaluation was conducted every 6 months [6]. The effect of training in CPR and AED on the self-perceived attitudes of health-care professionals to performing resuscitation was the subject of a study in two Swedish hospitals [7]. The results showed that health-care professionals—particularly nurses—had improvements in their attitudes to performing CPR and knowledge of CPR after training [7]. Factors that negatively influence the attitudes of nursing staff toward administration of CPR include fear of contracting a contagious illness [8–10] and lack of training, which might reduce confidence in performing CPR [8]. Similarly, health-care workers can be reluctant to provide mouth-to-mouth ventilation without the appropriate means [9, 10], because of anxiety that they might contract an illness [9, 10], that they might cause trauma [11] or that the AED might not work properly [11].

Assessment of attitudes, knowledge and influences that affect the use of CPR and AEDs among health-care professionals (physicians, nurses, emergency medical technicians and paramedics) is important. Attitudes to the performance of CPR vary between countries [12–14], but to our knowledge no previous study has been conducted in Saudi Arabia to determine the effect of CPR training on these attitudes. Our aim was to study the effect of CPR–AED training on the attitudes of health-care workers before and after attending a BLS-provider course, with the following objectives: [1] to assess health-care providers’ attitudes toward CPR–AED training before taking the course, [2] to identify factors influencing attitudes toward CPR–AED use, [3] to determine the effect of attending the BLS course on attitudes toward CPR and defibrillation and [4] to evaluate the level of retention of these attitudes among providers who had previously received training.

## Methods

### Study setting

The PTC was commissioned in 1998 and offers continuing-education courses on topics such as life support, critical care, trauma, obstetrics, ultrasonography and minimally invasive surgery. The PTC has a strong collaborative relationship with the AHA, and is affiliated with several other North American societies. The PTC is staffed with coordinators, administrators, a secretary, an accountant and a cashier. As preparation for accreditation and certification by the Joint Commission International, the PTC is able to certify all health-care providers at MNG-HA with BLS courses and advanced resuscitation programs.

### Study design

A quasi-experimental study was conducted. The experimental, post-BLS group (of individuals who took BLS courses within the 3 month study period) was given self-reported questionnaires after being examined for BLS certification. For the non-experimental, pre-BLS group, an assigned person distributed self-reported questionnaires to all health-care providers during their registration for any of the courses conducted at the PTC (including BLS courses) over the same period of 3 months, until the sample size was the same as that for the post-BLS group.

### Study subjects

Health-care professionals who could understand, read and speak English were candidates for the study. Layperson rescue workers, BLS instructors and those who had no previous BLS training were excluded.

### Sample-size calculation

Initially, a pilot study was conducted on 62 pre-BLS participants, and the mean attitude score was 70%, with a standard deviation of 15%. Based on these results, and to demonstrate an assumed post-BLS score of 74%, with 90% power and 95% confidence intervals, the calculated sample size requirements were 296 participants per group. A number of 500 participants were decided to include from each group to compensate for incomplete questionnaires or faulty entries. Thus, a total of 421 and 321 completed questionnaires were collected from the pre- and post-BLS groups respectively.

### Intervention

The BLS-provider course is 4–5 h in duration and covers scene safety, patient assessment, activation of the emergency-response system, chest compression, airway and breathing and the use of the AED to resuscitate an adult, child or infant who is not responding, not breathing and has no detectable pulse. Each topic is taught as a critical skill and is practiced separately. Thereafter, all the skills are performed in one demonstration. The most important skills are considered to be chest compression and early defibrillation, because of the strong evidence and consensus on their effectiveness, so considerable time is given to practicing chest-compression skills. The delivery format of the course instruction is a video demonstration followed by ‘practice-while-watching’. Instructors act as facilitators during skills practice and as evaluators when testing a candidate. Psychomotor and cognitive skills are tested at the end of the course. The psychomotor skills are check-listed, and providers are expected to pass all the steps of critical sequences for treating both adults and infants. Failure to correctly demonstrate a critical skill is given the designation ‘needs remediation’, requiring the participant to demonstrate the skill again until he or she passes. A written examination contains 25 multiple-choice questions with a single best answer for each, and a minimum score of 21 (84%) is required to pass the examination. BLS manuals are provided to participants when they register for a course, although evidence suggests that there is no difference in theoretical knowledge and skill retention between individuals who receive a manual 1 month prior to the course and those who do not [15]. All health-care providers in the post-BLS group were contacted by the end of the course and were asked to complete the questionnaires after providing consent.

### Data collection

A questionnaire was developed, of previously validated tool, to enable assessment of educational status, knowledge, attitude and factors that influence the use of CPR and AED [16–19]. This questionnaire encompassed three scales. The first scale consisted of 17 items pertaining to status of education and certification with respect to BLS, advanced cardiovascular life support and pediatric advanced life support, and their use and availability [16, 19]. The second scale consisted of 15 items pertaining to attitudes to the use of (and legislation relating to) CPR and AED [17, 18]. The third scale consisted of 12 items related to factors that influence the use of CPR and AED [19]. The questionnaires also included items relating to age, sex, residency, occupation and previous exposure to cardiac-arrest cases. The investigators were blinded to data collection, and were not directly involved with recruitment. To ensure feasibility and clarity, we performed a pilot study at the PTC with 62 health-care professionals.

### Data management

The Statistical Package for the Social Sciences software (SPSS version 21.0; IBM Corporation, Armonk, NY, USA) was used for data analysis. Both descriptive and analytic statistics were assessed. The chi-squared test was used for categorical data and the t-test was used for continuous numerical data. Pearson’s correlation was applied to test for significant correlations between the concern and attitude scores. A three-point Likert scale, ranging from 0 point “Disagree”, 1 point “Neutral” to 2 points “Agree” was applied, and for negative statements, the opposite score was applied. A scoring system assessed attitudes and concern regarding CPR and AED. We used 75% of total attitude score as a cut-off point to determine positive attitude [20], and used 50% of total concern score as a cut-off point to determine high concern, and this cut off point was equivalent to 75th percentile of concern score in this study. Multiple linear regression analyses were applied to identify the significant predictors of the attitude and concern scores. Statistical significance was considered at *p* < 0.05.

### Ethical considerations

Participation in this study was voluntary. HCWs were assured that their feedback would not affect their performance evaluations, work status or salaries. No written consent was sought, as there were no personal identifiers on the questionnaires, and this was approved by the IRB. Submission of responses to the questionnaire was considered to constitute implied consent. The voluntary nature of participating in the survey was made explicit and unambiguous in the cover letter. The investigators did not coerce or entice anyone to complete the questionnaire. Any participant could decline to return the questionnaire. The study was approved by the institutional review board (IRB) of the MNG-HA (Ref. # RC12/049).

## Results

### Participants’ characteristics

A total of 421 pre-BLS (56.7%) and 321 post-BLS (43.3%) healthcare providers were studied. The majority of pre-BLS and post-BLS groups were females (70.5% & 62.6%, *p* = 0.02), nurses (60.3% & 34.3%, *p* < 0.001), of medical specialty (69.4% & 61.1%, p = 0.02), and with more than 10 years of work experience (53.2% & 57.9%, *p* = 0.09), Table 1.

**Table 1:** Personal and BLS-related characteristics of the pre-BLS and post-BLS groups

	Pre-BLS n (%) 421 (56.7)	Post-BLS n (%) 321 (43.3)	Statistical analysis
Participants’ characteristics			
Gender			
Female	297 (70.5)	201 (62.6)	χ^2^ = 5.189, *p* = 0.02*
Male	124 (29.5)	120 (37.4)	
Education level			
Lower education *(BS/Diploma)*	375 (89.1)	250 (77.9)	χ^2^ = 17.177, *p* < 0.001*
Higher education*(MSN/PhD)*	46 (10.9)	71 (22.1)	
Job title			
Physicians	77 (18.3)	93 (29.0)	χ^2^ = 49.667, *p* < 0.001*
Nurses	254 (60.3)	110 (34.3)	
Other professionals	90 (21.4)	118 (36.7)	
Specialty			
Medical	292 (69.4)	196 (61.1)	χ^2^ = 5.572, *p* = 0.02*
Surgical	129 (30.6)	125 (38.9)	
Work Experience (years)			
0–5	114 (27.1)	85 (26.5)	χ^2^ = 2.504, *p* = 0.29
6–10	83 (19.7)	50 (15.6)	
> 10	224 (53.2)	186 (57.9)	
BLS-related characteristics			
Advanced life-support license			
None	263 (62.5)	220 (68.5)	χ^2^ = 2.949, *p* = 0.086*
ACLS/PACLS	158 (37.5)	101 (31.5)	
Exposure to cardiac arrest cases in the previous year			
0 times	101 (24.0)	124 (38.6)	χ^2^ = 20.230, *p* < 0.001*
1–9 times	243 (57.7)	139 (43.3)	
≥ 10 times	77 (18.3)	58 (18.1)	
Previous BLS training			
Once	64 (15.2)	65 (20.3)	χ^2^ = 18.923, *p* < 0.001*
Twice	74 (17.6)	90 (28.0)	
Three and above	283 (67.2)	166 (51.7)	

*Abbreviations*: *ACLS* advanced cardiovascular life support; *BLS* basic life support; *PALS* pediatric advanced life support, *statistical significance

### Previous experience with CPR and AED

The majority of pre-BLS and post-BLS participants had three or more previous BLS trainings (67.2% & 51.7%, p < 0.001), had been exposed to cardiac arrest cases at least once within the last year (76% & 61.4%, p < 0.001), yet had no advanced life support license (62.5% & 68.5%, *p* = 0.086), Table 1.

### Attitudes toward BLS

Participants in the pre-BLS and post-BLS groups responded to 15 statements assessing attitudes toward various aspects relating to CPR and AEDs, with a three-point scale ranging from “Agree” to “Disagree” (Table 2). Training was associated with an increase in the prevalence of positive attitudes, from 53.4% in the pre-BLS group to 64.8% in the post-BLS group (χ^2^ = 9.66, *p* = 0.002), with a rise in mean scores from 74.7% to 78.8% (*t* = 4.27, *p* < 0.001). The majority of participants in each group agreed that all clinics should be equipped with an AED, and indicated that they would support/participate in a community CPR/AED project, they thought AED should be mandated in clinics and office settings, they could perform CPR on their own, they were able to work as a member of a resuscitation team and, if an AED was available, they would use it to care for a patient with cardiac arrest. A minority of participants indicated that they thought the prognosis of resuscitated patients was poor, that only doctors should defibrillate and that defibrillation damages the heart.

**Table 2:** Attitude toward basic life support (BLS) training among health-care providers in pre-BLS (*n* = 421) and post-BLS (*n* = 321) groups

	BLS group	Disagree *n* (%)	Neutral *n* (%)	Agree *n* (%)	χ^2^	*p* value
Attitude (15 statements)						
1. I think CPR & use of AED should be rehearsed at least once per year	Pre	49 (11.6)	107 (25.4)	265 (63.0)	7.468	0.024*
	Post	42 (13.1)	108 (33.6)	171 (53.3)		
2. I am able to work as a member of a resuscitation team	Pre	33 (7.9)	116 (27.6)	271 (64.5)	9.578	0.008*
	Post	12 (3.8)	115 (35.9)	193 (60.3)		
3. I can perform CPR on my own	Pre	37 (8.8)	96 (22.9)	287 (68.3)	5.963	0.051
	Post	17 (5.3)	60 (18.8)	243 (75.9)		
4. Prognosis of resuscitated patient is poor	Pre	181 (43.5)	183 (44.0)	52 (12.5)	4.865	0.088
	Post	125 (39.7)	162 (51.4)	28 (8.9)		
5. Only doctors should defibrillate	Pre	220 (52.4)	101 (24.0)	99 (23.6)	20.152	<0.001*
	Post	213 (66.6)	69 (21.6)	38 (11.8)		
6. I know how to defibrillate	Pre	65 (15.4)	120 (28.5)	236 (56.1)	13.619	0.001*
	Post	23 (7.2)	85 (26.6)	211 (66.2)		
7. I think defibrillation damages a patient’s heart	Pre	294 (70.0)	112 (26.7)	14 (3.3)	0.748	0.688
	Post	231 (72.9)	77 (24.3)	9 (2.8)		
8. Defibrillation should be performed by any healthcare professional on the scene	Pre	96 (22.9)	96 (22.9)	227 (54.2)	8.253	0.016*
	Post	48 (15.0)	70 (21.9)	201 (63.1)		
9. I hesitate to use an AED because I fear damaging the patient	Pre	266 (63.3)	115 (27.4)	39 (9.3)	4.887	0.087
	Post	223 (70.8)	72 (22.9)	20 (6.3)		
10. If an AED is available, I would use it to attend a cardiac arrest patient	Pre	31 (7.4)	104 (24.8)	284 (67.8)	7.574	0.023*
	Post	12 (3.8)	64 (20.1)	242 (76.1)		
11. I am willing to attend an AED training course at my own expense	Pre	110 (26.2)	98 (23.3)	212 (50.5)	26.802	<0.001*
	Post	37 (11.6)	107 (33.6)	174 (54.8)		
12. I agree that all clinics should be equipped with an AED	Pre	8 (1.9)	36 (8.6)	376 (89.5)	4.870	0.088
	Post	1 (0.3)	35 (11.0)	283 (88.7)		
13. I would support/participate in community CPR/AED project	Pre	7 (1.7)	67 (16.0)	346 (82.3)	5.388	0.068
	Post	0 (0.0)	53 (16.6)	266 (83.4)		
14. I would perform mouth-to-mouth ventilation during CPR	Pre	150 (35.7)	146 (34.8)	124 (29.5)	10.707	0.005*
	Post	80 (25.1)	118 (37.0)	121 (37.9)		
15. I think AED should be mandated in the clinic and office settings	Pre	19 (4.5)	75 (17.9)	326 (77.6)	10.308	0.006*
	Post	2 (0.6)	54 (16.9)	263 (82.5)		
		Pre-BLS	Post-BLS			
Attitude score		*n* (%)	*n* (%)			
Positive (>75%)		225 (53.4)	208 (64.8)			
Negative/neutral (≤75%)		196 (46.6)	113 (35.2)	χ^2^ = 9.66, *p* < 0.001		
Mean ± SD		74.7 ± 13.8	78.8 ± 12.5	*t* = −4.269, *p* < 0.001		

*Abbreviations*: *AED* automated external defibrillator; *BLS* basic life support; *CPR* cardiopulmonary resuscitation, *statistical significance

Figures shown in the table are for available data only

**Fig. 1:**
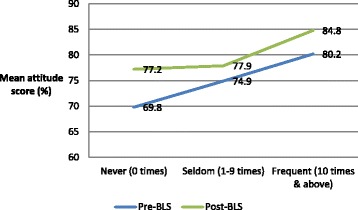
Association between attitude scores and the number of previous exposures to cardiac-arrest cases in individuals in the pre-BLS and post-BLS groups. Abbreviation: BLS, basic life support

Figure 1 shows the association between the pre-BLS and post-BLS attitude scores and the number of exposures to cardiac-arrest cases in the preceding year. In both groups, attitude scores increased significantly with an increasing number of exposures (*p* < 0.001).

**Fig. 2:**
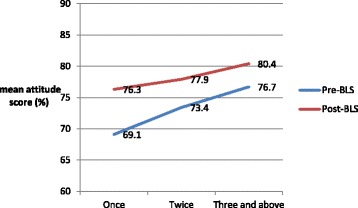
Association between attitude score and number of BLS training courses attended prior to the study period. Abbreviation: BLS, basic life support

Figure 2 shows the association between the pre-BLS and post-BLS attitude scores and the number of previous BLS training courses. In both groups, attitude scores increased significantly with an increasing number of previous training courses (*p* < 0.001).

### Concern about BLS

Participants in the pre-BLS and post-BLS groups responded to 10 statements assessing concern about various aspects relating to CPR and AEDs, with a three-point scale ranging from “Agree” to “Disagree.” High overall levels of concern were seen in 36.3% of participants in the pre-BLS group and 37.1% of the post-BLS group (Table 3). The main concerns reported by the participants were lack of training, lack of self-confidence, lack of legal coverage, knowing the victim and fear of contracting a disease. Fewer participants indicated concerns with treating a young victim, fear of further harming a heart-attack victim, treating a patient of a different gender, treating a patient of the same gender or treating a victim from a different geographic area.

**Table 3:** Concern regarding basic life support (BLS) among health-care providers in pre-BLS (*n* = 421) and post-BLS (*n* = 321) groups

Concern (10 statements)	BLS group	Disagree *n* (%)	Neutral *n* (%)	Agree *n* (%)	χ^2^	*p* value
1. I think that the fear of further harming a heart attack victim affects me	Pre	202 (48.3)	139 (33.0)	77 (18.4)	8.454	0.015*
	Post	119 (39.4)	132 (3.7)	51 (16.9)		
2. I believe that lack of self-confidence influences me in initiating CPR	Pre	150 (35.7)	115 (27.4)	155 (36.9)	2.287	0.319
	Post	105 (34.7)	98 (32.3)	100 (33.0)		
3. I fear that my lack of training influences me greatly in initiating CPR	Pre	123 (29.3)	127 (30.2)	170 (40.5)	1.337	0.513
	Post	93 (30.7)	100 (33.0)	110 (36.3)		
4. I believe that a young victim positively affects my decision to initiate CPR	Pre	205 (48.8)	119 (28.3)	96 (22.9)	4.762	0.092
	Post	126 (41.9)	107 (35.5)	68 (22.6)		
5. I think a victim being of a different gender affects me	Pre	299 (71.2)	64 (15.2)	57 (13.6)	13.856	0.001*
	Post	185 (61.1)	80 (26.4)	38 (12.5)		
6. I think a victim being from a different geographic area affects me	Pre	291 (69.3)	79 (18.8)	50 (11.9)	7.175	0.028*
	Post	183 (60.6)	81 (26.8)	38 (12.6)		
7. I think that lack of legal coverage greatly affects me	Pre	180 (43.0)	100 (23.9)	139 (33.1)	17.462	<0.001*
	Post	107 (35.3)	116 (38.3)	80 (26.4)		
8. I think that my fear of contracting a disease affects me	Pre	178 (42.4)	130 (31.0)	112 (26.6)	3.927	0.140
	Post	113 (37.3)	115 (38.0)	75 (24.7)		
9. I believe that if I know the victim, this will positively affect my decision	Pre	187 (44.7)	94 (22.5)	137 (32.8)	4.654	0.098
	Post	124 (41.2)	89 (29.6)	88 (29.2)		
10. I think that a victim being of the same gender will affect my decision to initiate CPR	Pre	288 (68.7)	75 (17.9)	56 (13.4)	10.268	0.006*
	Post	175 (58.1)	82 (27.2)	44 (14.7)		
		Pre-BLS	Post-BLS			
Concern score		*n* (%)	*n* (%)			
High (≥50%)		153 (36.3)	119 (37.1)			
Low (<50%)		268 (63.7)	202 (62.9)		χ^2^ = 0.042, *p* = 0.84	
Mean ± SD		37.4 ± 24.6	39.4 ± 22.9		*t* = −1.089, *p* = 0.28	

*Abbreviations*: *BLS* basic life support; *CPR* cardiopulmonary resuscitation, *statistical significance

Figures shown in the table are for available data only

**Fig. 3:**
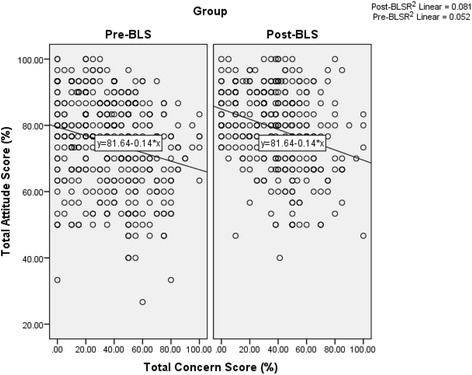
Correlation between concern scores (%) and attitude scores (%) relating to CPR/AED before and after BLS training. Abbreviations: AED, automated external defibrillator; BLS, basic life support; CPR, cardiopulmonary resuscitation

Figure 3 shows significant negative correlations between concern and attitudes scores in both the pre-BLS (*r* = −0.229, *p* < 0.001) and post-BLS (*r* = −0.284 *p* < 0.001) groups.

Table 4 shows the results of the linear regressions of concern and attitude scores for BLS with a number of covariates. Both the concern and attitude scores were predicted by the recent completion of a BLS training course. Previous BLS training was positively correlated with participants’ attitude toward BLS and negatively correlated with concern, indicating that training makes participants think positively about BLS, and not dwell on any possible negative aspects of providing treatment. Previous exposure to cardiac-arrest cases also had a positive effect on attitude. Physicians had concern scores >10% lower than nurses. Additionally, the concern score significantly decreased with increasing work experience. A high concern score was a significant predictor of a low attitude score.

**Table 4:** Linear regression analysis of BLS concern and attitude scores with independent variables

	β	S.E.	*t* value	Adjusted *p* value
BLS training (post-BLS vs. pre-BLS)				
Attitude	5.15	1.18	4.38	<0.001*
Concern	4.87	2.19	2.22	0.027*
Sex (male vs. female)				
Attitude	1.54	1.55	0.99	0.320
Concern	2.96	2.89	1.02	0.308
Education (higher vs. lower)				
Attitude	1.71	1.69	1.01	0.315
Concern	−1.72	3.18	−0.54	0.590
Job category (physicians vs. nurses)				
Attitude	−0.88	1.68	−0.52	0.601
Concern	−10.45	3.11	−3.37	0.001*
Exposure to cardiac arrest cases (yes vs. no)				
Attitude	3.44	1.45	2.38	0.018*
Concern	−3.74	2.70	−1.38	0.167
Previous BLS training (*n*)				
Attitude	2.10	0.79	2.64	0.008*
Concern	−3.99	1.48	−2.69	0.007*
Work experience (years)				
Attitude	−0.05	0.07	−0.64	0.520
Concern	−0.29	0.14	−2.19	0.029*
Concern (mean % score)				
Attitude	−0.09	0.02	−4.01	<0.001*
Constant	70.11	2.73	24.66	
	53.05	4.56	11.63	

*Abbreviation*: *BLS* basic life support, *statistical significance

## Discussion

In BLS training, the focus has been mainly on the knowledge and skills of initial life-support care. However, in real situations, attitude is also very important. We focused particularly on factors influencing the willingness to perform CPR, and evaluated them by administering questionnaires to health-care workers with more or less recent BLS training. In general, the effect of recent completion of BLS training on attitude was obvious, with mean scores of 74.7% pre-BLS and 78.8% post-BLS (*p* < 0.001). This finding is in agreement with the results of studies in college students in Japan [21] and in health-care workers in Sweden [22]. In our study, the proportion of health-care workers who reported a positive attitude increased significantly from the pre-BLS group to the post-BLS group, demonstrating a short-term positive effect of BLS training. This effect of recent BLS training on attitude remained after adjusting for known possible confounders, including previous BLS training. In regression analyses, recent BLS training predicted positive attitude, as did the number of previous BLS training courses, previous exposure to cardiac-arrest cases and low concern scores.

Although recent BLS training improved attitude scores, it also resulted in a greater level of concern with regard to performing CPR. This apparently contradictory finding is similar to observations in other studies [13, 21, 23–25], and might be explained by the fact that those with more knowledge about resuscitation could be more likely to cite fear of disease transmission as an obstacle to performing BLS, whereas their counterparts with less knowledge of BLS might not be aware of this possibility [26].

High concern scores were significant predictors of low attitude scores in our study. Higher levels of concern were reported with regard to lack of training and low self-confidence than other issues, which is consistent with the findings of a previous study [8]. Lack of legal coverage was another concern, which is also consistent with the literature [21].

Microorganisms that pose an infection risk during CPR include HIV [27], *Staphylococcus aureus* [28], and herpes simplex virus [29]. Fear of disease transmission was one of the main concerns in our study, as it has been in previous studies in countries including Norway [26], Japan [24], USA [13], Australia [30] and Hong Kong [31]. However, we found that the level of concern relating to the infection risk during CPR was not significantly different in the pre-BLS and post-BLS groups. This result differs from the findings of a study in Sweden [9] in which fears of infection while performing mouth-to-mouth ventilation were significantly reduced in both physicians and nurses by BLS training.

Repeating training helps staff to retain knowledge of CPR and the use of an AED [8]. Retention of knowledge and skills during and after training in CPR is difficult and requires systematic training with appropriate methodology [32]. In our study, the number of previous BLS training events attended had a positive effect, reducing concern scores and increasing attitude scores. In a previous study of CPR skills of professional nurses, assessed at regular intervals, re-education was found to enhance knowledge and skills [33]. Similarly, lack of training was reported as a main factor that influenced the attitudes of health-care providers toward the performance of CPR in a number of previous studies [8, 34–36]. The evidence suggests that training is important even for experienced staff [37].

Health-care providers can witness cardiac arrest in the clinic or during house calls, or even outside of work. In our cohort, the majority of participants reported witnessing cardiac arrest at least once within the preceding year. In both the pre-BLS and post-BLS groups, attitude scores were positively associated with the number of cardiac-arrest cases witnessed in the preceding year.

Every member of a health-care organization should be able to perform CPR and defibrillation [38]. In a cardiac-arrest situation, a doctor is expected to work as a leader of the resuscitation team [39]. Overall, the concern scores of physicians in our cohort were >10% lower than those of nurses. We also observed that work experience had the beneficial effect of reducing levels of concern. In a previous study [40], newly qualified doctors expressed a need for more instruction in working as a team leader. These results suggest that BLS training is beneficial, but that its effectiveness might be further improved by targeting specific aspects of training to specific occupations, and by addressing the different levels of concern that are associated with particular situations.

### Strengths and limitations

The strength of this study is the large sample size. Limitations include the subjective nature of the questionnaire responses, and the possibility of systematic bias in the sampling method [9]. Respondents may not recall exactly the number of previous exposure to cardiac arrest cases, and this might be a source of recall bias. Moreover, pre- and post-BLS groups were non-comparable in regard to some characteristics such as; job title, gender, educational level and number of previous BLS trainings, and this difference between the two groups of participants might be a source of selection bias. However, all such differences were adjusted, using the multiple linear regression analyses, to determine the predictors of BLS concern and attitude scores.

## Conclusions

Our results, in agreement with the results of previous studies, demonstrated that repeated educational programs can improve attitudes toward CPR performance and the use of AEDs. The number of previous BLS training courses attended was correlated with the levels of both attitude and concern, so that individuals with more training experience had better attitudes and less concern with regard to CPR and AEDs than individuals with limited experience.

Systematic and recurring training in CPR is important. Health-care organizations and individual providers should both be involved in ensuring that such courses are undertaken and completed, to optimize confidence in the performance of CPR, and potentially to save lives.

Future studies are necessary to assess the effects of different frequencies and formats of BLS training on performance in emergency settings.

## Abbreviations

AEDs: Automated external defibrillators
AHA: American Heart Association
BLS: Basic life-support
CPR: Cardiopulmonary resuscitation
IRB: Institutional Review Board
KAMC: King Abdulaziz Medical city
MNG-HA: Ministry of National Guard-Health Affairs
OHCA: Out-of-hospital cardiac arrest
PMS: percentage mean score
PTC: Postgraduate Training Center
SHA: Saudi Heart Association
